# U-Shaped Relationship Between Fibrinogen Level and 10-year Mortality in Patients With Acute Coronary Syndrome: Prospective Cohort Study

**DOI:** 10.2196/54485

**Published:** 2024-06-07

**Authors:** Yi ming Li, Yuheng Jia, Lin Bai, Bosen Yang, Mao Chen, Yong Peng

**Affiliations:** 1 Department of Cardiology West China Hospital Sichuan University Sichuan Province, Chengdu City China

**Keywords:** fibrinogen, acute coronary syndrome, 10-year mortality, risk factor, coronary artery disease, myocardial, heart disease, inflammatory factor, retrospective study, Kaplan-Meier analysis, mortality, all-cause mortality, cubic-spline curve, regression model

## Abstract

This study demonstrated that fibrinogen is an independent risk factor for 10-year mortality in patients with acute coronary syndrome (ACS), with a U-shaped nonlinear relationship observed between the two. These findings underscore the importance of monitoring fibrinogen levels and the consideration of long-term anti-inflammatory treatment in the clinical management of patients with ACS.

## Introduction

Identification of risk factors is essential in patients with coronary artery disease. As an important inflammatory factor and a key participant in coagulation, fibrinogen has attracted attention in the management of acute coronary syndrome (ACS). Previous research has also demonstrated that as an acute-phase protein, the levels of fibrinogen and its degradation products are higher in patients with COVID-19 than in the healthy population [1]. However, the relationship between fibrinogen levels and the prognosis of patients with ACS remains controversial [2]. Our previous study found that fibrinogen was an independent risk factor for mortality during the follow-up in patients with coronary artery disease [3]. In the ACS subgroup, no correlation was found between fibrinogen levels and mortality. In addition, the quantitative relationship between fibrinogen levels and the risk of mortality across a longer follow-up period needs further clarification.

## Methods

### Overview

We performed a prospective, large-scale, single-center study (Chinese Clinical Trial Registry ChiCTR 2100049313) to investigate the effect of fibrinogen levels on ACS prognoses. Patients were grouped according to the 5 quantile levels of plasma fibrinogen at admission. We used Kaplan-Meier analysis to estimate the cumulative incidence of all-cause mortality. The predictive value of fibrinogen levels for 10-year mortality was estimated by a Cox proportional hazards regression model. The impact of fibrinogen level on 10-year mortality was assessed by a restricted cubic spline (RCS) curve, which was derived from an adjusted Cox proportional hazards regression model.

### Ethical Considerations

The study protocol was approved by the institutional review board of West China Hospital in accordance with the Declaration of Helsinki (approval number 2012(243)). Written informed consent was obtained from all participants before enrollment in the study. Patients who come to the hospital for follow-up will be reimbursed for their transportation expenses.

## Results

**Figure 1:**
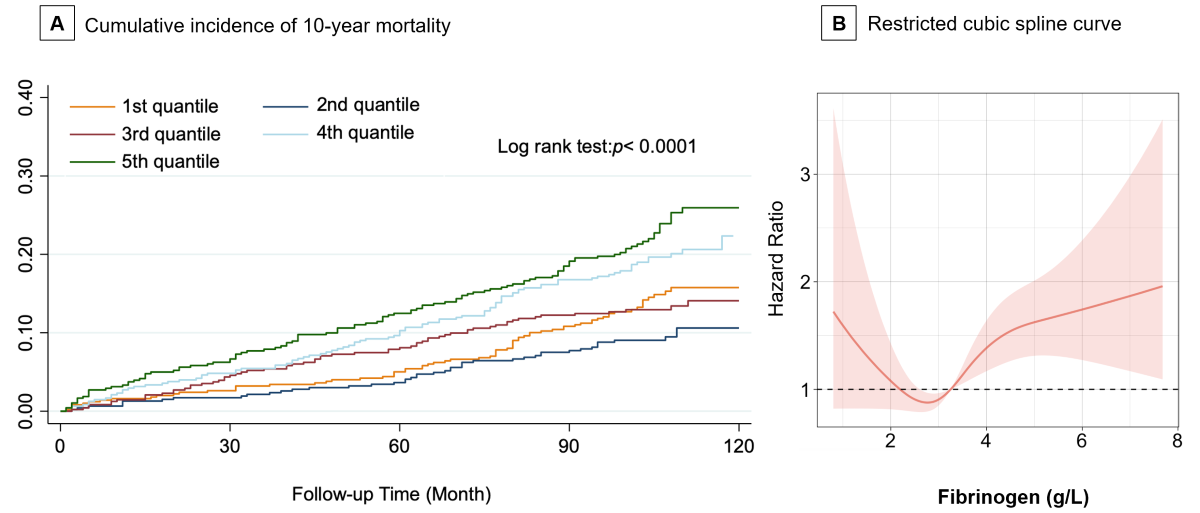
Cumulative incidence and adjusted hazard ratio for 10-year all-cause mortality. (A) Kaplan-Meier curves show a cumulative incidence of all-cause mortality at 10 years after it was stratified by 5 quantiles of plasma fibrinogen levels at admission. (B) The restricted cubic-spline curve shows the adjusted hazard ratio and its 95% confidence interval for 10-year all-cause mortality based on the plasma fibrinogen level. The *P* value for nonlinearity was <.001.

A total of 2434 consecutive patients with ACS who were admitted to West China Hospital in the Sichuan province of China, between December 10, 2010, and December 31, 2012, were enrolled in this study. After exclusion of 29 patients with in-hospital mortality, 2405 patients were included in the analysis. The mean age of the study population was 64.3 (SD 11.1) years. Plasma fibrinogen levels of these patients were normally distributed (skewness/kurtosis tests: *P*<.001), with a mean level of 3.41 (SD 1.06) g/L. The median follow-up time was 104 (IQR 98-111) months. In total, 387 (16.1%) patients died during the follow-up period. Kaplan-Meier analysis revealed that the cumulative incidence of mortality was significantly different among the 5 groups (log-rank test: *P*<.001; Figure 1A). Group 2 (fibrinogen level: 2.63-3.04 g/L) had the lowest incidence of mortality. In the Cox univariate regression analysis, fibrinogen was identified as a risk factor for 10-year mortality (hazard ratio [HR]: 1.24 [1.15-1.35]; *P*<.001). After adjusting for cardiovascular risk factors, fibrinogen was still found to be an independent risk factor (HR: 1.18 [1.08-1.29]; *P*<.001). The fibrinogen category in the multivariate Cox model, with group 2 serving as the referent, showed a nonlinear trend to mortality risk (Table S1 in Multimedia Appendix 1). Subgroup analysis suggested that in the ST-segment elevation myocardial infarction (STEMI)/non-STEMI (NSTEMI) subgroups, statistical results for fibrinogen showed the same trend (Table S2 in Multimedia Appendix 1).

The knot positions of the RCS analysis were set as the quartiles of fibrinogen levels. The RCS curve suggested strong U-shaped relationships between the adjusted risk of 10-year mortality and fibrinogen level, which was in accordance with the trend noted in the Kaplan-Meier analysis. The risk of all-cause mortality decreased until the fibrinogen level reached 2.81 g/L; it then started to increase thereafter (*P* value for nonlinearity <.001).

## Discussion

Acute-phase proteins are often associated with patient prognosis. Previous epidemiologic studies have suggested that elevated fibrinogen levels may mediate plaque rupture via prothrombotic or proinflammatory processes [2]. However, a lower fibrinogen level may indicate a worse general physical condition or may reveal hidden coagulation disorders in patients with ACS [4]. In this study, the long-term follow-up across nearly a decade and the quantitative RCS analysis suggested that fibrinogen levels at admission have a significant impact on the long-term prognosis of patients with ACS. The mechanisms underlying this phenomenon suggest that fibrinogen acts as both an acute-phase protein and a subclinical inflammatory indicator [5].

The U-shaped relationship between fibrinogen levels and 10-year mortality suggest the need for long-term anti-inflammatory or metabolism treatment, as well as the need to determine fibrinogen levels in patients with ACS. In addition, the nonlinear correlation between fibrinogen levels and the long-term mortality may restrict the effect of fibrinogen in some risk models that were derived from extended linear regression. The predictive value of fibrinogen needs to be estimated in nonlinear functions or by using machine learning classifiers in further studies.

## Appendix

Multimedia Appendix 1 Multivariate Cox model and subgroup analysis.

## Abbreviations

ACS: acute coronary syndrome
HR: hazard ratio
RCS: restricted cubic spline
STEMI: ST-segment elevation myocardial infarction

## Data Availability

The anonymized data set of this cohort can be requested by contacting the corresponding author via email.
