# Supplementary material for: U-Shaped Relationship Between Fibrinogen Level and 10-year Mortality in Patients With Acute Coronary Syndrome: Prospective Cohort Study
Source: JMIR Public Health Surveill. 2024 Jun 7;10:e54485. doi: 10.2196/54485 (PMC11212677; doi:10.2196/54485)
Supplement: Multimedia Appendix 1 [file publichealth_v10i1e54485_app1.docx]

Supplementary 1: Multivariate Cox model with the fibrinogen category group 2serving as referent.

| Group | Hazard ratio | 95% Confidence interval | P value |
| --- | --- | --- | --- |
| 1 | 1.76 | 1.20-2.57 | 0.004 |
| 2 | Ref | Ref | Ref |
| 3 | 1.40 | 0.95-2.01 | 0.092 |
| 4 | 1.83 | 1.27-2.63 | 0.001 |
| 5 | 2.31 | 1.62-3.30 | <0.001 |

Adjusted with age (per decade), sex, hypertension, diabetes, serum creatine level, LDL-C level, and Killip class.

Supplementary 2: Multivariate Cox model with the fibrinogen category group 2serving as referent in subgroup analysis.

| Group | Hazard ratio | 95% Confidence interval | P value |
| --- | --- | --- | --- |
| STEMI/NSTEMI subgroup | | | |
| 1 | 1.80 | 1.04-3.10 | 0.035 |
| 2 | Ref | Ref | Ref |
| 3 | 1.66 | 0.97-2.87 | 0.065 |
| 4 | 2.31 | 1.40-3.81 | 0.001 |
| 5 | 2.52 | 1.55-4.09 | <0.001 |
| Unstable angina subgroup | | | |
| 1 | 1.70 | 1.00-2.87 | 0.050 |
| 2 |  |  |  |
| 3 | 1.09 | 0.62-1.92 | 0.765 |
| 4 | 0.95 | 0.52-1.76 | 0.878 |
| 5 | 0.93 | 0.44-1.99 | 0.856 |

Adjusted with age (per decade), sex, hypertension, diabetes, serum creatine level, LDL-C level, and Killip class.
